# Synergistic Inhibition of Colorectal Cancer Growth by Combined PI3K and COX-2 Blockade in Cell Lines and Patient-Derived Organoids

**DOI:** 10.3390/pharmaceutics18060683

**Published:** 2026-05-30

**Authors:** Emily Nghiem, Ariel Tzamarot, Terence Li, Zimo Huang, Mahshid Mohammadi, Dior Dedushi, Yvonne Saenger, Fernand Bteich, Chaoyuan Kuang

**Affiliations:** 1 Department of Surgery, Albert Einstein College of Medicine, and Montefiore Medical Center, Bronx, NY 10467, USA; emily.nghiem@einsteinmed.edu; 2 Montefiore Einstein Comprehensive Cancer Center, Albert Einstein College of Medicine, Bronx, NY 10461, USA; ariel.tzamarot@einsteinmed.edu (A.T.); terence.li@einsteinmed.edu (T.L.); zimo.huang@einsteinmed.edu (Z.H.); mahshid.mohammadi@einsteinmed.edu (M.M.); dior.dedushi@einsteinmed.edu (D.D.); yvonne.saenger@einsteinmed.edu (Y.S.); fbteich@montefiore.org (F.B.); 3 Department of Molecular Pharmacology, Albert Einstein College of Medicine, Bronx, NY 10461, USA; 4 Department of Microbiology and Immunology, Albert Einstein College of Medicine, Bronx, NY 10461, USA; 5 Department of Oncology, Albert Einstein College of Medicine, Bronx, NY 10461, USA

**Keywords:** colorectal cancer, organoids, celecoxib, inavolisib, PI3Kα, COX-2, *PIK3CA*, preclinical study

## Abstract

**Background/Objectives:** PI3K/AKT/mTOR is a key pathway in cell proliferation, metabolism, and survival. Activating *PIK3CA* mutations are seen in up to 20% of colorectal cancers and are associated with increased cyclo-oxygenase-2 (COX-2) expression. Recent studies demonstrated a significant survival benefit from taking low-dose aspirin, a nonselective COX inhibitor, supporting further exploration of the synergistic effects of combined PI3Kα inhibitor (inavolisib) and COX-2 inhibitor (celecoxib) therapy. **Methods:** The effects of celecoxib–inavolisib combination treatment were tested on human colorectal cancer cell lines and patient-derived organoid models. Experiments included cell viability and colony formation assays, immunoblotting, and immunofluorescence. **Results:** We found that celecoxib and inavolisib demonstrated synergy in suppressing the growth of colorectal cancer cell lines, grown in both 2D and 3D cell culture, regardless of *PIK3CA* mutation status. In patient-derived organoid models, while synergy was seen in both organoids, growth of the *PIK3CA* mutated organoid was more potently suppressed. Immunoblotting of cells after combination treatment showed decreased expression of mitogenic signaling marker p-AKT across all 2D cell lines and in both cell lines grown as 3D spheroids, as well as increased expression of apoptotic marker cPARP in four out of five 2D cell lines and in both cell lines grown as 3D spheroids. Immunofluorescence staining of organoids after combination treatment, however, showed no significant increase in expression of apoptotic marker Cas-3 nor in mitogenic marker Ki-67 in either organoid. Furthermore, an apoptosis assay performed on two cell lines showed no significant increase in Annexin V or phosphatidylserine staining. **Conclusions:** Celecoxib and inavolisib demonstrated synergy in suppressing the growth of both colorectal cancer cell lines and patient-derived organoids, though *PIK3CA* mutation status did not appear to affect drug efficacy in cell lines as it did in patient-derived organoids. Potential compensatory or resistance mechanisms might include oncogene drivers in the MAPK/ERK pathway. When compared to monotherapy, combination therapy was the only drug condition to significantly increase the percentage of apoptotic cells based on Annexin V and phosphatidylserine staining, and this effect was only seen in the *PIK3CA* mutated cell line. Ultimately, our findings provide preliminary support for celecoxib–inavolisib combination treatment as a rational therapeutic avenue warranting further preclinical investigation.

## 1. Introduction

PI3K/AKT/mTOR is a prominent pathway involved in the proliferation, metabolism, and survival of cells. There are three classes of phosphoinositide 3-kinases (I, II, III). Class I PI3Ks are heterodimers comprising one of four catalytic p110 subunits (α, β, δ, or γ) and a regulatory subunit (p85). The catalytic subunits are encoded by the *PIK3CA*, *PIK3CB*, *PIK3CD*, and *PIK3CG* genes, respectively. Surface receptor tyrosine kinases (RTKs) such as EGFR, HER2, and IGF1R are activated by the binding of their ligands. RTKs then phosphorylate adaptor proteins which in turn bind to the p85 regulatory subunit of PI3K, leading to activation of PI3K and its downstream effector AKT. Activated KRAS protein can also interact with and activate PI3K. Finally, PI3K phosphorylates PIP2 into PIP3, which recruits and activates AKT [1].

Activating mutations in *PIK3CA* are among the most common oncogenic alterations in human cancers, particularly seen in hormone receptor (HR)-positive, HER2-negative breast cancer (40%) [2], colon cancers (15–20%) [3], diffuse large B-cell lymphomas (8%) [4], and head and neck squamous cell carcinomas (30.5%) [5]. For colorectal cancer (CRC) in particular, the most common activating *PIK3CA* gene mutations involve exons 9 and 20 [3].

Targeting PI3K has proven challenging over the years. Attempts, particularly in lymphoma and chronic lymphocytic leukemia, have largely been unsuccessful and associated with high mortality rates. Potential challenges include the limited oncogenic driver role of mutant *PIK3CA*, suboptimal patient stratification in clinical trials, drug-associated adverse effects, and compensatory feedback upregulation of alternative signaling pathways upon PI3K inhibition [6]. Idelalisib, an inhibitor of leukocyte-enriched PI3Kδ initially approved for relapsed follicular and small lymphocytic lymphomas, was withdrawn from the market in 2022 due to serious adverse events, most notably intestinal perforation. Umbralisib, which also targets PI3Kδ, met a similar fate. In breast cancer, however, multiple agents have achieved approval, including alpelisib and inavolisib, which are given in combination with endocrine therapy and cyclin-dependent kinases (CDK) 4/6 inhibitors. Both drugs are alpha-selective PI3K inhibitors, and the most common associated adverse events include hyperglycemia, stomatitis, and cytopenias [7]. Inavolisib is a novel, significantly more selective inhibitor that targets only PI3Kα. It inhibits PI3K signaling and induces degradation of the mutant p110α protein through an RTK-dependent mechanism, achieving sustained pathway suppression [8]. In the INAVO120 phase III clinical trial, inavolisib, in combination with the selective estrogen receptor degrader fulvestrant and the CDK4/6 inhibitor palbociclib, significantly improved progression-free survival in patients with *PIK3CA*-mutant breast cancer when compared to placebo with palbociclib and fulvestrant (mPFS 15.0 vs. 7.3 months, HR 0.43; 95% CI, 0.32 to 0.59; *p* < 0.001) [9]. These results led to the approval of inavolisib, in combination with fulvestrant and palbociclib, as first-line treatment in the setting of metastatic HR+/HER2− breast cancer with a qualifying *PIK3CA* mutation [10].

*PIK3CA* mutations have also emerged as predictive biomarkers for response to cyclo-oxygenase-2 (COX-2) inhibitors, particularly in CRC. COX-2 is an inducible enzyme involved in inflammation and tumor progression through the production of prostaglandin E2 (PGE2), which sustains survival signaling and suppresses anti-tumor immunity [11]. Studies in CRC have shown that patients with *PIK3CA* mutations express higher levels of COX-2 and gain a significant survival benefit from COX-2 inhibition with agents such as celecoxib and nonsteroidal anti-inflammatory drugs such as acetylsalicylic acid (aspirin) [12]. The phase III CALGB/SWOG 80702 trial had a preplanned subset analysis to test if adding celecoxib to standard adjuvant chemotherapy could reduce the risk of recurrence and improve survival in patients with stage III resected colon cancer and *PIK3CA* gain-of-function mutations. In this study, celecoxib significantly improved the overall survival (OS) for patients with *PIK3CA* mutations (HR 0.44 [95% CI, 0.22 to 0.85] compared to 0.94 [95% CI, 0.68 to 1.30] for no celecoxib) [11]. Most recently, in 2024, the phase III ALASCCA trial studied the use of adjuvant low-dose aspirin in CRC patients with somatic mutations in the PI3K pathway. The recurrence rate was reduced by nearly 50% for patients with *PIK3CA*-mutated CRC who took aspirin for 3 years when compared with placebo (95% confidence interval [CI], 0.24 to 0.98; *p* = 0.04) [13,14].

Co-targeting of PI3Kα and COX-2 is an attractive prospect and may represent a synergistic therapeutic approach. Combining inavolisib, which selectively depletes p110α, with COX-2 inhibitors that suppress inflammatory and pro-survival prostaglandin signaling could potentially achieve enhanced tumor suppression of *PIK3CA*-mutant cancers.

## 2. Materials and Methods

### 2.1. Cell Lines

Human CRC cell lines (HCT116, HT29, DLD1, RKO, and SW480) and the murine cell line L-WRN were obtained from American Type Culture Collection (ATCC). ATCC numbers for each cell line are listed in Supplemental Table S1. HCT116, HT29, DLD1, RKO, and SW480 were cultured in RPMI 1640 (Corning^®^, Corning, NY, USA; 10-040-CV) supplemented with 10% fetal bovine serum (FBS) (Avantor^®^, Randor, PA, USA; 97068-069) and 1% penicillin-streptomycin while L-WRN was cultured in DMEM (Corning^®^, 10-013-CV) supplemented with 10% FBS and 1% penicillin-streptomycin. All cell lines were maintained at 5% CO_2_ and 37 °C and grown with 100 U/mL penicillin, and 100 μg/mL streptomycin. For the cell culture of 3D spheroids, SW480 and DLD1 were cultured in RPMI 1640 supplemented with 5% Matrigel^®^ (Corning^®^, 354234), 3% FBS, and 1% penicillin-streptomycin. The media contained 10 ng/mL of EGF (Gibco^®^, Grand Island, NY, USA; PHG0313), 10 ng/mL of bFGF, and 2 µg/mL of heparin. All experiments were conducted using cell lines at passages 2-15. All cell lines were monitored for *Mycoplasma* infection with Myco-Sniff-Valid™ (MP Biomedicals™, Irvine, CA, USA; 093050301) and, if positive, *Mycoplasma* treatment was performed with Plasmocure™ (InvivoGen, San Diego, CA, USA; ant-pc) until infection was undetectable prior to conducting experimentation. All cell lines underwent HLA/STR analysis and were confirmed against vendor datasheets prior to experimental use.

### 2.2. Patient Specimens

All patients were consented and registered on the IRB-approved Montefiore Einstein Comprehensive Cancer Center Biobank protocol (Einstein IRB# 2021-13730) before tissue and data acquisition. Clinical recruitment, coordination, and regulatory assistance were provided by the Cancer Clinical Trials Office (CCTO) of the Montefiore Einstein Comprehensive Cancer Center (MECCC). The study of patient specimens and patient-derived models was conducted under an IRB approved protocol (Einstein IRB# 2025-16811). Tissues were obtained from patients who underwent standard-of-care surgical resection after gross tumor specimens were examined by pathology lab staff. Specimens were inspected, divided, and portions were used immediately to (1) fix in 10% neutral buffered formalin, (2) establish patient-derived organoids (PDOs), and (3) freeze in DMEM supplemented with 20% FBS and 10% Dimethyl Sulfoxide (DMSO) (Fisher BioReagents™, Fair Lawn, NJ, USA; BP231-100). Formalin specimens were fixed for 24 h (± 1 h), transferred into 70% ethanol, and submitted to the Albert Einstein Histology and Comparative Pathology Facility for Formalin-Fixed, Paraffin-Embedded (FFPE) processing, tissue sectioning, and H&E staining.

### 2.3. Patient-Derived Organoids

The PDO named CRC 23-006 was established as part of the Montefiore Einstein Cancer Center Biobank. Biobank participants were identified by treating physicians and/or surgeons, consented, and enrolled in the biobank. Fresh tissue was then collected for investigation from CRC surgical resections. Approximately 0.5 cm fragments of tumor tissue were used for the establishment of PDOs. Tumor tissue fragments were mechanically and chemically disrupted using a razor blade and diluted Gentle Collagenase/Hyaluronidase (Stemcell Technology, Vancouver, BC, Canada; MSPP-07919), respectively. Tumor clusters were embedded in Matrigel^®^ and surrounded by complete PDO media which was supplemented with WRN conditional media as well as ROCK inhibitor Y-27632 (MedChemExpress, Monmouth Junction, NJ, USA; HY-10071). All PDOs used were passaged at least twice and grown with a doubling time of 3-10 days. The PDO named CK 10278 is also known as National Cancer Institute (NCI) PDO 362531-283 R-V1 and was obtained from NCI Patient-Derived Models Repository, received frozen in DMSO and media, thawed, and grown as previously described [15]. The growth medium used for all PDOs followed NCI protocols and methods.

### 2.4. Drug Treatment

All treatments were performed by diluting either a DMSO drug stock or DMSO without drug (control) into the aqueous growth media. Drug-free growth media was then removed and replaced with drug-containing media at the indicated concentrations. For PDO treatment, domes of Matrigel^®^ and PDOs were left intact while drug-free media was exchanged for drug-media, as described above. Cell cultures of 3D spheroids were grown in suspension, drug-free growth media was left in the wells, and new drug-containing media was added to achieve the concentrations indicated in the corresponding figures.

### 2.5. Cell Viability Assays

**Crystal violet:** After treatment, cells were rinsed with Hanks’ Balanced Salt Solution (HBSS) (Gibco™, 14025-092), fixed in 1% crystal violet (Fisher BioReagents™, BP231-100) in 10% neutral buffered formalin for 10 min, and then destained with tap water. Finally, the cells were air dried and imaged.

**Cell line viability:** Cells were plated in 96-well plates and placed in a 5% CO_2_, 37 °C incubator for 24 h to allow for attachment. Next, they were treated with various drug conditions. Finally, cells were assayed 48 h later using a chemical viability assay (MTS, Promega CellTiter 96™ Aqueous One Solution Cell Proliferation Assay, Madison, WI, USA; G3581). For cell culture of 3D spheroids, cells were plated in coated 96-well plates and were allowed to grow and form spheres for 3–5 days. They were then drugged as described previously for 48 h. Viability was measured using CellTiter-Glo™ 3D Cell Viability Assay (Promega, G9683). All viability assays were measured using the FLUOstar Omega microplate reader (BMG Labtech, Ortenberg, Germany). All assays were performed with technical triplicates.

**PDO viability:** PDOs were grown to confluency then digested and filtered through a 40 µm filter to eliminate large tumor clusters and debris. They were then resuspended in fresh Matrigel^®^ and plated in 10 µL aliquots per well in a 96-well plate. PDOs were allowed to grow and re-establish for 3–5 days, followed by drug treatment for 5 days. Viability was measured using CellTiter-Glo™ 3D assay. All viability assays were measured using the FLUOstar Omega microplate reader. All assays were performed with technical triplicates.

### 2.6. Colony Formation Assay

Colony formation was assayed by plating equal numbers of 24 h drug-treated cells in 6-well plates at appropriate dilutions with no drug, followed by visualization and quantification of colonies by crystal violet staining after 12–14 days (refer to crystal violet method above). Analysis of colony-formation units was performed using ImageJ (version 2.16.0/1.54p). Images were converted to 8-bit, and the threshold was set to the default red with no black background, with the upper and lower threshold bars set to 0 and 200, respectively. The “Process” function was then applied, followed by selecting “Binary”, setting as binary, and applying “Watershed” to separate clumped cell line colonies. Individual colonies were generated using “Analyze Particles,” and the count values were recorded. These data were then input into GraphPad Prism (version 10.6.1) to generate bar graphs.

### 2.7. Immunoblotting

Protein was collected from cell lines grown in 6-well plates after treatment with the indicated drug conditions. Cells were washed and then digested using 100 µL of a cocktail of RIPA (Boston Bioproducts, Ashland, MA, USA; NC9193720) supplemented with Halt™ Protease Inhibitor Cocktail (Thermo Scientific™, Waltham, MA, USA; 78340), Phosphatase Inhibitor Cocktail 2 (Millipore Sigma, Burlington, MA, USA; P5726), and Phosphatase Inhibitor Cocktail 3 (Millipore Sigma, P0044). Cells were incubated on ice for 10 min then scraped and pipetted into 1.7 mL centrifuge tubes and sonicated (FisherScientific, 422-A) for 5 s on and 5 s off for a total 15 s at 20% AMP. The lysate was cleared by centrifugation at maximum speed (16,000× *g*) for 10 min, followed by collection of the supernatant and discarding of the pellet. Lysate protein concentration was quantified using Pierce™ 660 nm Protein Assay Reagent (FisherScientific, 22660) with BSA standards. Lysates were mixed with 4× Laemmli Sample Buffer (Bio-Rad Laboratories, Inc., Hercules, CA, USA; 1610747) with 2-Mercaptoethanol (Millipore Sigma, M7522) and heated at 85 °C for 10 min. The final lysates were then loaded into 4–15% Mini-PROTEAN^®^ TGX™ Precast gels (Bio-Rad Laboratories, Inc., 4561086EDU) alongside Precision Plus Protein™ Dual Color Standards ladder (Bio-Rad Laboratories, Inc., 1610374EDU). Gels were run with 1× running buffer (Bio-Rad Laboratories, Inc., 1610772) at 160 volts for 35–45 min. Protein was transferred from the gels onto activated Immobilon^®^-P PVDF Membranes (Millipore Sigma, IPVH00010) using a Trans-Blot^®^ Turbo™ Transfer System (Bio-Rad Laboratories, Inc., 1704150) and 1× transfer buffer (Bio-Rad Laboratories, Inc., 1610771), methanol, and 10% sodium dodecyl sulfate (Lonza, Basel, Switzerland; 51213).

Membranes were blocked for 1 h in 1× TBS (Bio-Rad Laboratories, Inc., 1706435) and Tween20 (TBST) (TCI 9005-64-5) supplemented with 10% milk Blotting-Grade Blocker (Bio-Rad Laboratories, Inc., 1706404). Blocked membranes were incubated overnight at 4 °C with primary antibody diluted in TBST containing 10% milk, with gentle rocking. Membranes were washed three times with TBST for 6 min each and then incubated with HRP-conjugated secondary antibody in TBST + 10% milk for 1 h at room temperature. Clarity Western ECL Substrate solution (Bio-Rad Laboratories, Inc.,1705061) was mixed fresh and added to the membranes, followed by imaging in a Li-Cor Odyssey FC (Li-Cor Biosciences, Lincoln, NE, USA). Western blot images were analyzed using ImageStudio (version 6.1). All images were calibrated to the same background value using ImageJ (version 2.16.0/1.54p). The optical densities (OD) of the protein bands were then analyzed with the gel analyzer function and area under the curve quantification in ImageJ. Individual marker values were normalized to ß-actin values in Excel. Ratios and other normalizations were also processed in Excel. The final data were computed and presented in bar graphs in GraphPad Prism (version 10.6.1), expressed as fold change relative to control, which was set as 1. Primary antibodies are listed in Supplemental Table S2.

### 2.8. Light Microscopy

All brightfield and phase contrast micro-photographs were obtained using the Echo Revolve 4 combination inverted/upright microscope (Echo) at 10× magnification.

### 2.9. Statistical Analyses

Statistical analyses were carried out using GraphPad Prism (version 10.6.1). Data were analyzed by one-way ANOVA assuming Gaussian distribution. Variance equality was assessed by Brown–Forsythe test. Where variances were unequal, Welch’s ANOVA was applied. Where appropriate, Šídák’s test and Dunnett’s test were applied for multiple comparisons. Dunnett’s test was used to assess for statistically significant differences in results between drug conditions as compared to control, whereas Šídák’s test was used to assess for statistically significant differences in results between various drug conditions.

### 2.10. CompuSyn

Combination Index Scores were generated with CompuSyn (version 1.0), based on viability values obtained from MTS assays for cell lines and CellTiter-Glo™ 3D assays for organoids, along with the corresponding drug concentrations.

### 2.11. Immunofluorescence

CRC 23-006 and CK 10278 PDOs were cultured to confluency, then digested and passed through a 40 µm filter to remove large tumor clusters and debris. They were then resuspended in fresh Matrigel^®^ and plated in 40 µL aliquots per well of chamber slides. The samples were then drugged with celecoxib (12.5 µM), inavolisib (1.25 µM), or a combination of both for 48 h, alongside untreated organoids as controls. Each sample was then washed to remove the Matrigel^®^ and fixed in 4% Paraformaldehyde (PFA) (Paraformaldehyde, Thermo Scientific Chemicals, 047377-9L). After fixation, the organoids were labelled with the apoptotic marker cleaved caspase-3 (Cas-3) (Biotium, Fremont, CA, USA; NucView^®^ Caspase-3 Enzyme Substrates, excitation 488 nm, emission green, 1:200 dilution) and the proliferation marker Ki-67 (Abcam, Cambridge, UK; 15580, excitation 647 nm, emission far-red, 1:200 dilution), then mounted using a DAPI-containing medium (ProLong™ Diamond Antifade Mountant, Invitrogen P36962, excitation 405 nm, emission blue). Immunofluorescence images were acquired using a Nikon CSU-W1 Spinning Disk Confocal microscope (40× objective) and analyzed with Imaris (version 10.2.0) software. Images were analyzed manually by two reviewers. The highest-quality images with adequate staining penetrance and minimal background were selected from each set of technical triplicates. ImageJ was used to mark and keep track of cell counts. Total cells in each organoid were identified and counted first by examining DAPI staining. Ki-67 and Cas3 staining were then examined, respectively. Cells were deemed positive if demonstrating fluorescence in over 50% of the cell, as determined by a unanimous decision between both reviewers.

### 2.12. Flow Cytometry

SW480 and DLD1 cells were cultured and treated in four conditions: control, celecoxib monotherapy, inavolisib monotherapy, and combination therapy at the indicated concentrations for 48 h. A flow cytometry apoptosis assay was then performed using Annexin V and phosphatidylserine (PS) staining (BD Annexin V: PE Apoptosis Detection Kit I, 559763) to complete three technical triplicates. Flow cytometry analysis was completed on Invitrogen Attune NxT Flow Cytometer and analyzed with Flowjo (version 10). Values were then entered into GraphPad Prism (version 10.6.1) to create bar graphs.

## 3. Results

### 3.1. Celecoxib and Inavolisib Demonstrate Synergistic Effects in Suppressing the Growth and Survival of Colorectal Cancer Cell Lines in 2D Cell Culture

**Figure 1:**
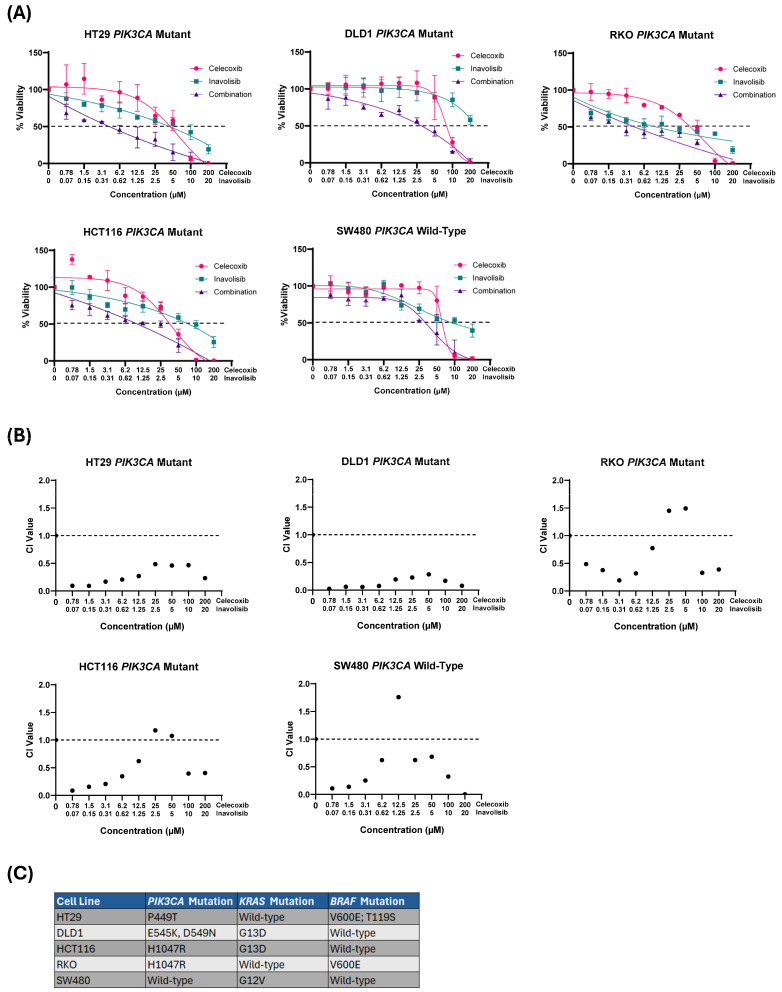

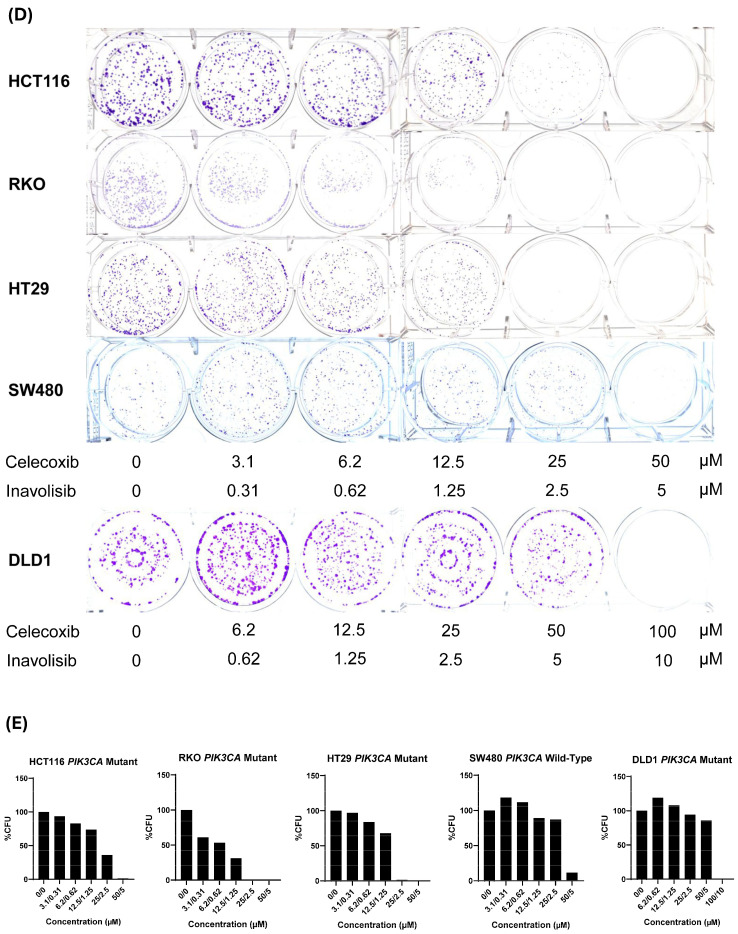
(**A**) Chemical viability assay performed 48 h after cell line drugging using single-agent celecoxib, inavolisib, and celecoxib/inavolisib combination treatments. IC_50_ is indicated with the dotted line. (**B**) Combination index scores calculated from results of cell line drugging. Points below the dotted line (<1) indicate synergy. (**C**) Table of cell line *PIK3CA* mutation status. (**D**) Colony formation unit assay performed 12–14 days after replating cells drugged for 24 h with celecoxib/inavolisib combination treatment. (**E**) Quantification of colony formation unit assay.

To assess synergy against CRC cell lines, celecoxib–inavolisib combination treatments at varying concentrations were compared with single-agent treatments across five cell lines (HCT116, HT29, DLD1, RKO, and SW480) using cytotoxicity assays (Figure 1A). Chemical viability assays were carried out 48 h after treatment. The results showed a more potent suppression of cell growth and survival with combination treatment as compared to monotherapy across all cell lines, regardless of *PIK3CA* mutation status (Figure 1C). In all cell lines, the IC_50_ of the combination treatment was lower than the IC_50_ of each respective drug when used as monotherapy. This effect was also present regardless of *PIK3CA* mutation status. To quantify the synergistic effects of the combination treatment in Figure 1A, CompuSyn (version 1.0) software was used to calculate combination index (CI) scores with the Chou-Talalay method (Figure 1B). Cell lines HT29 and DLD1 demonstrated synergy (CI < 1) at all nine drug concentrations, SW480 demonstrated synergy at eight out of nine drug concentrations, while HCT116 and RKO both demonstrated synergy at seven out of nine drug concentrations. Concurrently, a colony formation unit (CFU) assay was performed to visualize the effects of combination treatment on CRC cell growth (Figure 1D). Cells were treated with varying concentrations of combination treatment and, 24 h later, were re-plated in 6-well plates without any drug. Crystal violet staining was performed 12 to 14 days later. In all the cell lines, apart from the lowest drug concentration condition for DLD1 and SW480, colony formation was progressively suppressed with increasing drug concentration. These results were further confirmed with CFU quantification (Figure 1E).

### 3.2. Celecoxib and Inavolisib Demonstrate Varying Synergistic Effects in Suppressing the Growth and Survival of Colorectal Cancer Cell Lines in 3D Cell Culture

**Figure 2:**
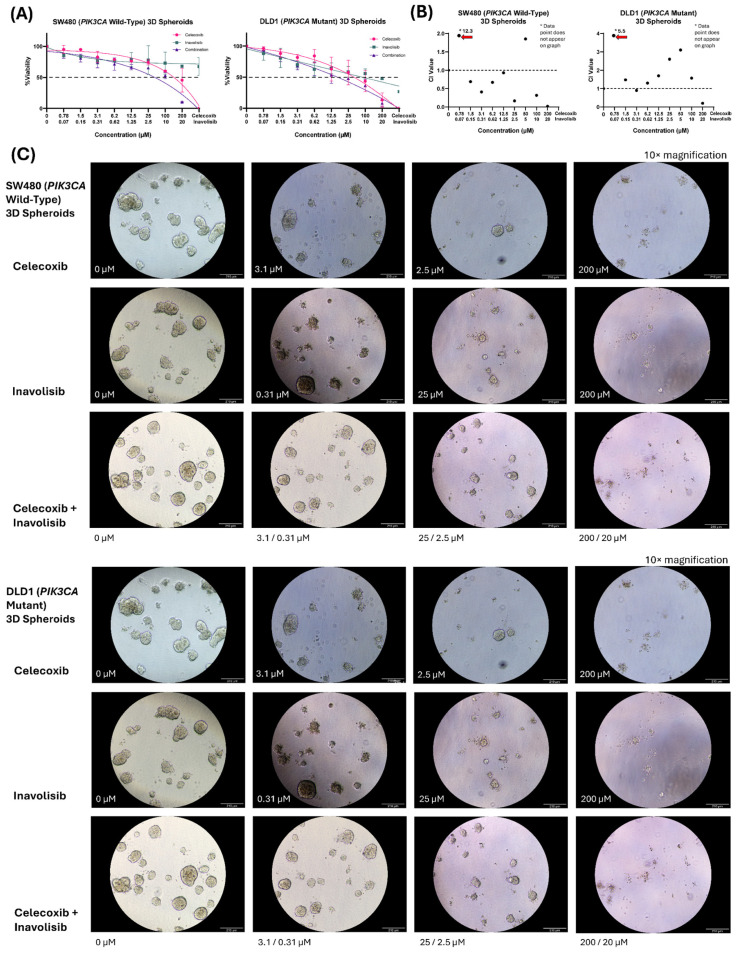
(**A**) Chemical viability assay performed 48 h after spheroid drugging using single-agent celecoxib, inavolisib, and celecoxib/inavolisib combination treatments. IC_50_ is indicated with the dotted line. (**B**) Combination index scores calculated from results of spheroid drugging. Points below the dotted line (<1) indicate synergy. (**C**) Brightfield images at 10× magnification of spheroids 48 h after drugging with single-agent celecoxib, inavolisib, and celecoxib/inavolisib combination treatments.

To explore whether drug effects varied across 2D and 3D cell culture, one *PIK3CA* wild-type cell line (SW480) and one *PIK3CA* mutated cell line (DLD1) were chosen for further experimentation. Cells were cultured to form 3D spheroids and drugged in an identical experimental set-up as previously described for 2D cell culture. Viability was measured using cytotoxicity assays (Figure 2A) and, similarly, CI scores were calculated (Figure 2B). Prior to performing cytotoxicity assays, brightfield images were taken at 10× magnification to visualize the effects of celecoxib–inavolisib combination treatment on 3D spheroids (Figure 2C). As seen in 2D cell culture, the IC_50_ of the combination treatment was lower than the IC_50_ of each respective drug when used as monotherapy in both cell lines, regardless of *PIK3CA* mutation status. Discrepancies in CI scores were present, however, with SW480 demonstrating synergy at six out of nine drug concentrations and DLD1 demonstrating synergy at only two out of nine drug concentrations (Figure 2B).

### 3.3. Celecoxib–Inavolisib Combination Treatment Suppresses AKT Phosphorylation in Colorectal Cancer Cell Lines in Both 2D and 3D Cell Culture

**Figure 3:**
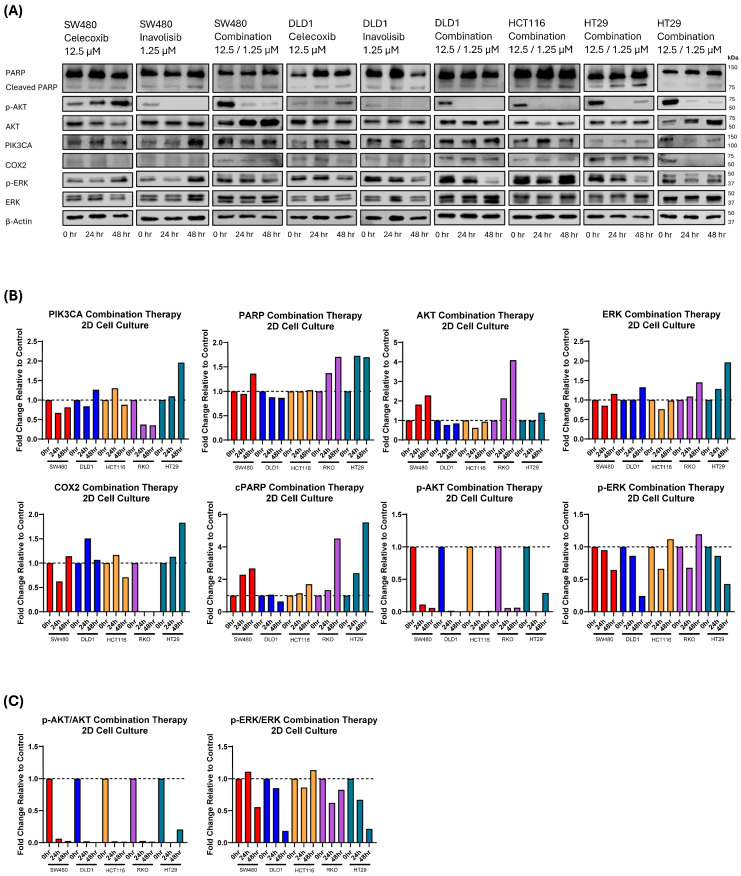

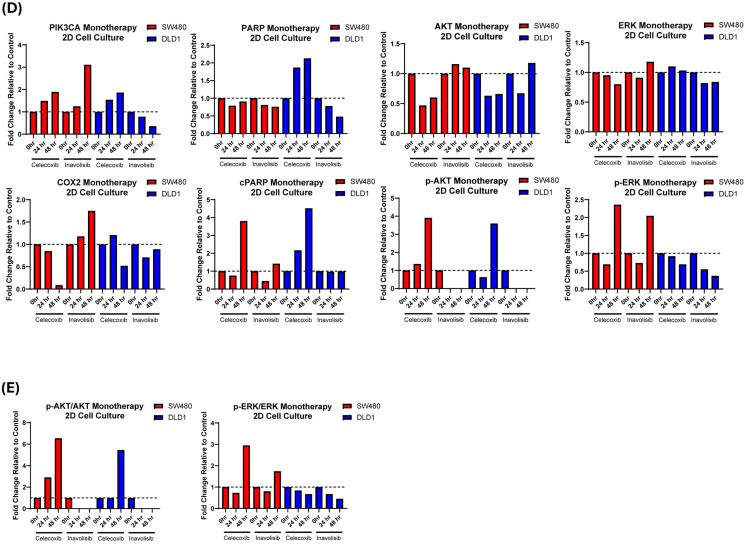
(**A**) Western blots of cell lysate collected at 0, 24, and 48 h after celecoxib/inavolisib combination treatment; Western blots of cell lysate collected at 0, 24, and 48 h after celecoxib and inavolisib monotherapy (SW480 and DLD1). (**B**) Quantification of results of combination therapy cell line Western blots expressed as fold change relative to control (dotted line at Y = 1). (**C**) p-AKT/AKT and p-ERK/ERK ratios calculated from results of combination therapy cell line Western blot quantification. (**D**) Quantification of results of monotherapy cell line Western blots expressed as fold change relative to control (dotted line at Y = 1). (**E**) p-AKT/AKT and p-ERK/ERK ratios calculated from results of monotherapy cell line Western blot quantification.

Considering results from the first two experiments, we next aimed to elucidate the mechanism of synergy between celecoxib and inavolisib. Western blots were performed on lysates from five CRC cell lines (HCT116, HT29, DLD1, RKO, and SW480) at 0, 24, and 48 h after celecoxib–inavolisib combination treatment (Figure 3A). These results were further confirmed by quantification and normalization to ß-actin expression, graphed as fold change relative to control (Figure 3B). The results demonstrated decreasing AKT phosphorylation (measured as p-AKT) across all five cell lines and decreasing ERK phosphorylation (measured as p-ERK) in SW480, HT29, and DLD1. These changes were reflected in the p-AKT/AKT and p-ERK/ERK ratios (Figure 3C). PARP cleavage, measured as cleaved PARP (cPARP), increased in all cell lines except DLD1, which showed an initial increase followed by a decrease at 48 h. AKT, ERK, and PARP did not demonstrate any consistent, time-dependent trends in levels of protein expression across the various cell lines.

To better investigate the effects of both inhibitors on signal transduction, Western blots were performed on SW480 and DLD1 lysates in a similar manner after treatment with celecoxib and inavolisib monotherapy (Figure 3A) with results confirmed by quantification and normalization to ß-actin expression, graphed as fold change relative to control (Figure 3D). Discernible trends with celecoxib monotherapy included increasing *PIK3CA* expression in both cell lines, decreasing COX2 expression in SW480, increasing PARP expression in DLD1, increasing PARP cleavage in DLD1, decreasing ERK expression in SW480, and decreasing ERK phosphorylation in DLD1. Discernible trends with inavolisib monotherapy included increasing *PIK3CA* expression in SW480 but decreasing *PIK3CA* expression in DLD1, decreasing PARP expression in both cell lines, decreasing AKT phosphorylation in both cell lines, and decreasing ERK phosphorylation in DLD1. Ratios of p-AKT/AKT and p-ERK/ERK were calculated (Figure 3E) with celecoxib monotherapy resulting in an increase in p-AKT/AKT ratio in SW480 and a decrease in p-ERK/ERK ratio in DLD1, while inavolisib monotherapy resulted in a decrease in p-AKT/AKT ratio in both cell lines and a decrease in p-ERK/ERK ratio in DLD1.

**Figure 4:**
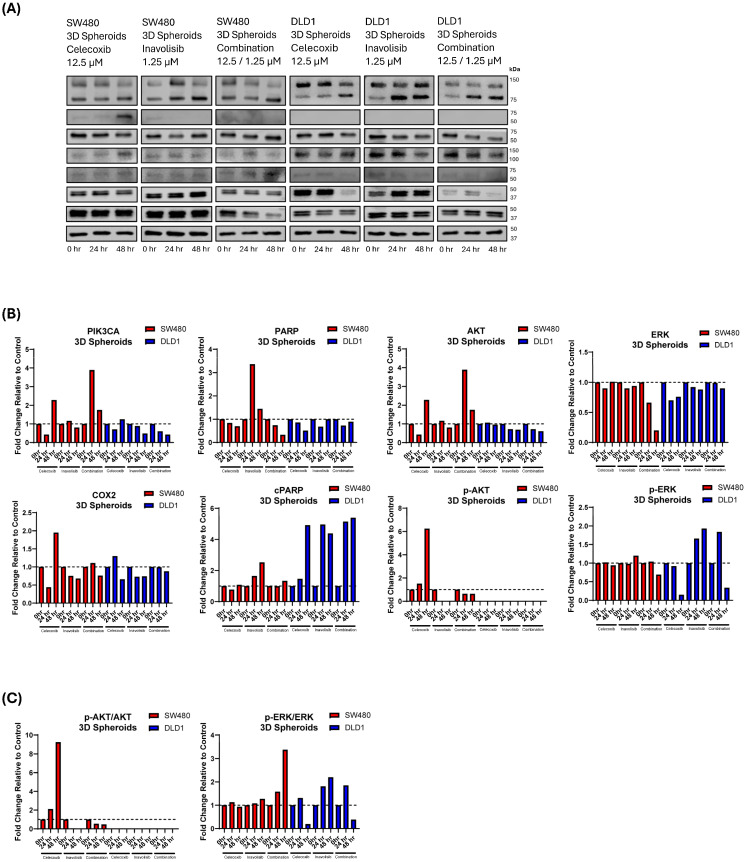
(**A**) Western blots of lysate from spheroids collected at 0, 24, and 48 h after celecoxib and inavolisib monotherapy and celecoxib/inavolisib combination treatment. (**B**) Quantification of results of spheroid Western blots expressed as fold change relative to control (dotted line at Y = 1). (**C**) p-AKT/AKT and p-ERK/ERK ratios calculated from results of spheroid Western blot quantification.

The same experimental set-up was used to perform Western blots on SW480 and DLD1 grown as 3D spheroids, testing the effects of celecoxib and inavolisib monotherapy as well as combination therapy (Figure 4A). Results were confirmed by quantification and normalization to ß-actin expression, graphed as fold change relative to control (Figure 4B). Ratios for p-AKT/AKT and p-ERK/ERK were also calculated (Figure 4C). While there were not many distinct time-dependent trends in protein expression nor many drug effects consistent across both cell lines, the most notable trends included increasing p-ERK/ERK ratio across both cell lines with inavolisib monotherapy, decreasing p-AKT/AKT ratio across both cell lines with inavolisib monotherapy and combination treatment, and increasing cPARP levels across both cell lines with combination therapy.

### 3.4. Celecoxib and Inavolisib Demonstrate Synergistic Effects in Suppressing the Growth and Survival of Colorectal Cancer Patient-Derived Organoids

**Figure 5:**
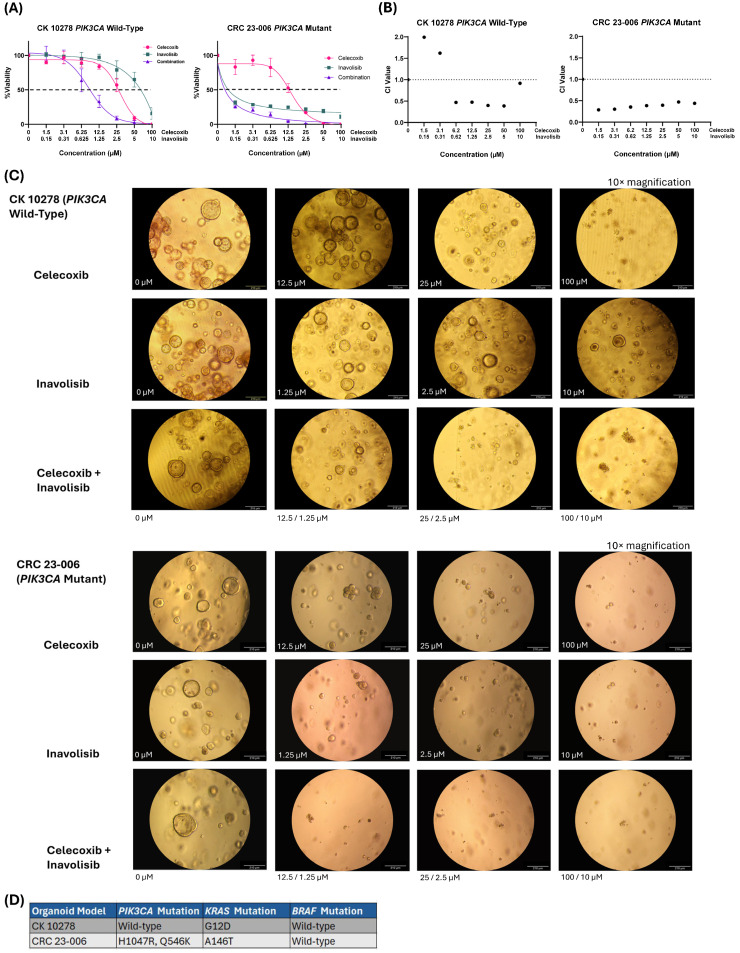
(**A**) Chemical viability assay performed 5 days after patient-derived organoid drugging using single-agent celecoxib, inavolisib, and celecoxib/inavolisib combination treatments. IC_50_ is indicated with the dotted line. (**B**) Combination index scores calculated from results of patient-derived organoid drugging. Points below the dotted line (<1) indicate synergy. (**C**) Brightfield images at 10× magnification of patient-derived organoids 5 days after drugging with single-agent celecoxib, inavolisib, and celecoxib/inavolisib combination treatments. (**D**) Table of patient-derived organoid *PIK3CA* mutation status.

To assess synergy against CRC PDOs, celecoxib–inavolisib combination treatment was compared at varying concentrations with single-agent treatment in two PDO models (CK 10278 and CRC 23-006) using cytotoxicity assays (Figure 5A). Chemical viability assays were carried out 5 days after treatment. The results showed more potent suppression of PDO growth and survival with combination treatment as compared to monotherapy. Unlike the lower but comparable IC_50_ values seen in CRC cell lines, *PIK3CA* mutant CRC 23-006 had notably lower IC_50_ values for inavolisib monotherapy and combination therapy when compared to the *PIK3CA* wild-type PDO CK 10278 (Figure 5D).

Prior to performing cytotoxicity assays, brightfield images were taken at 10× magnification to visualize the effects of combination treatment on PDO growth (Figure 5C). In the images of CK 10278, it was visually apparent that the organoids treated with combination therapy were smaller and demonstrated more dissociation when compared to those treated with monotherapy. These effects were seen across all drug concentrations, especially in comparison to inavolisib monotherapy. The images of CRC 23-006 followed a similar trend, with evidence of even more drastic growth suppression seen starting even at the lowest drug concentration. These results were further confirmed by CI scores calculated from the data in Figure 5A. CK 10278 demonstrated synergy at five out of seven drug concentrations while CRC 23-006 demonstrated synergy at all seven drug concentrations (Figure 5B).

### 3.5. Celecoxib–Inavolisib Combination Treatment Shows Varying Effects on Ki-67 and Cas-3 Staining in Colorectal Cancer Patient-Derived Organoids

**Figure 6:**
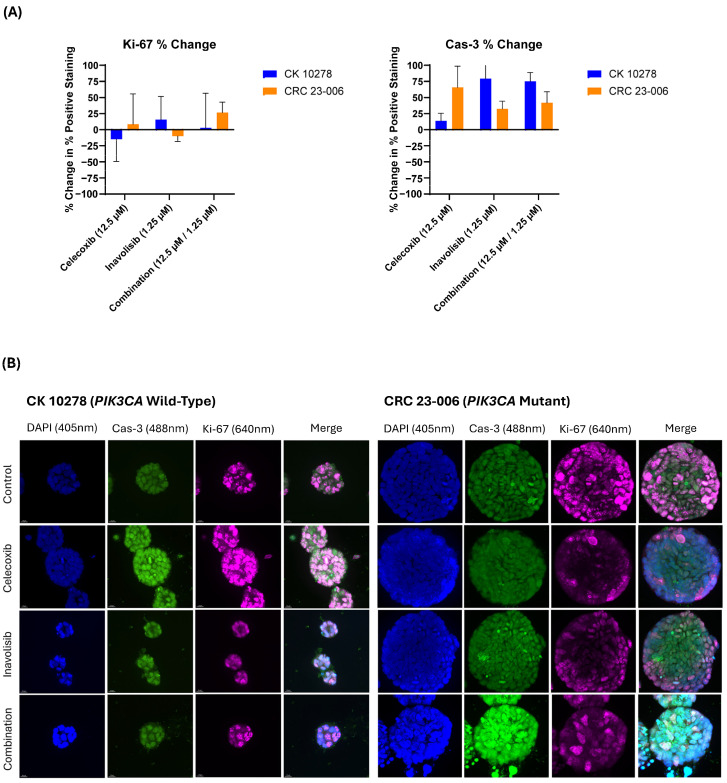
(**A**) Quantification of Ki-67 and Cas-3 activity in patient-derived organoids 48 h after drugging with single-agent celecoxib, inavolisib, and celecoxib/inavolisib combination treatment expressed as percent change relative to control. (**B**) Immunofluorescence images taken 48 h after patient-derived organoid drugging using single-agent celecoxib, inavolisib, and celecoxib/inavolisib combination treatments. Panels show DAPI, Cas-3, Ki-67, and merged staining, respectively.

To further characterize the synergistic effects of celecoxib–inavolisib combination treatment on CRC PDO growth, immunofluorescence (IF) staining was performed (Figure 6B). CK 10278 and CRC 23-006 PDOs were treated under four conditions: control, celecoxib monotherapy, inavolisib monotherapy, or combination therapy. Western blot analysis of 2D cell lines following combination treatment showed suppressed p-AKT (a marker of mitogenic signaling) in all five cell lines and increased cPARP (a marker of apoptosis) in four out of five cell lines. Western blot analysis of cell lines grown as 3D spheroids showed a similar decrease in AKT phosphorylation and increase in PARP cleavage in both cell lines following combination treatment. Accordingly, we examined the effects of combination treatment on PDO proliferation and apoptosis using Ki-67 and Cas-3 staining, respectively. After 48 h of drug treatment, IF staining was performed for DAPI, Ki-67, and Cas-3, followed by quantification of Ki-67 and Cas-3 signals expressed as percent change relative to control (Figure 6A). These results demonstrated no clear trend in effect on Ki-67 activity for either PDO model, whereas Cas-3 activity was increased for all three drug conditions across both PDO models.

### 3.6. Celecoxib–Inavolisib Combination Treatment Shows Varying Effects on Annexin V Binding in Colorectal Cancer Cell Lines

**Figure 7:**
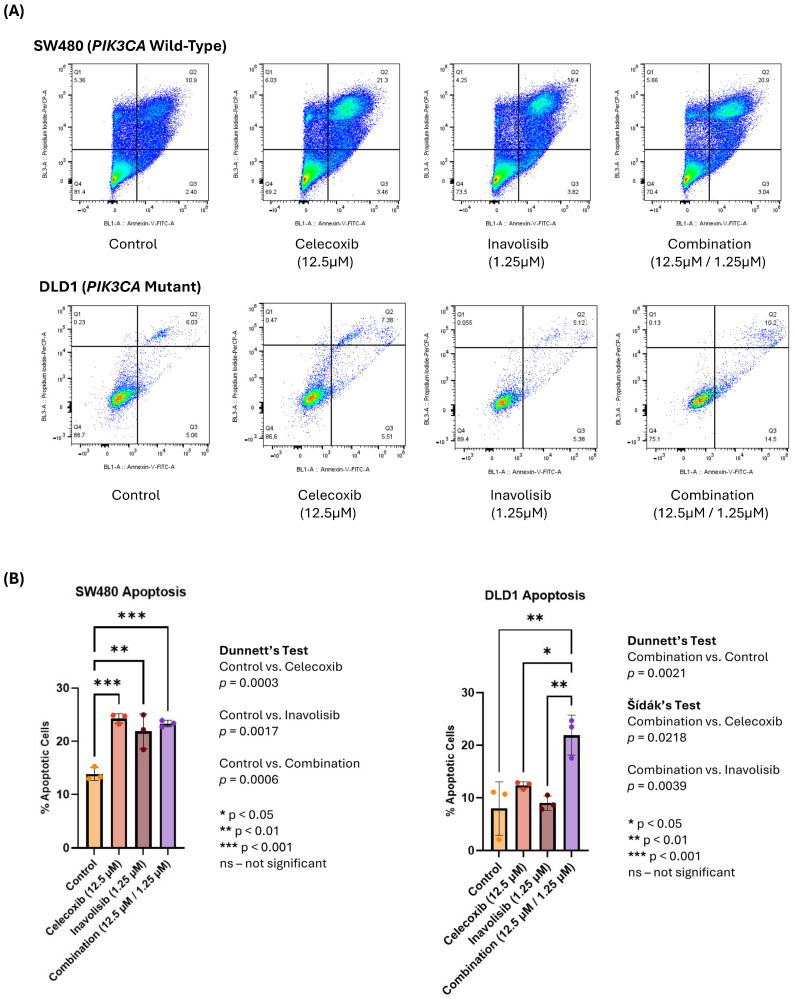
(**A**) Flow cytometry of cells 48 h after drugging with single-agent celecoxib, inavolisib, and celecoxib/inavolisib combination treatment staining for Annexin V and phosphatidylserine. (**B**) Quantification of the results of flow cytometry expressed as percentage of apoptotic cells. Comparisons of statistical significance are annotated with *p*-values listed at right.

To investigate the effects of celecoxib–inavolisib combination treatment on apoptotic pathways in CRC cell lines, an apoptosis assay was performed on SW480 and DLD1 cells using flow cytometry to assess Annexin V and phosphatidylserine (PS) staining (Figure 7A). Drug conditions included control, celecoxib monotherapy, inavolisib monotherapy, and combination therapy at the indicated concentrations for a duration of 48 h. The results from Figure 7A were then quantified and expressed as percentage of apoptotic cells (Figure 7B). For SW480 combination treatment resulted in the second highest percentage of apoptotic cells, while for DLD1 combination treatment resulted in the highest percentage of apoptotic cells.

## 4. Discussion

Given the frequency of COX-2 overexpression and *PIK3CA* mutations seen in CRC, we hypothesized that dual inhibition of COX-2 and PI3Kα could synergistically increase anti-tumor effects against in vitro CRC models. We tested celecoxib–inavolisib combination therapy in five different CRC cell lines grown in 2D cell culture with various *PIK3CA* mutations (Figure 1C). Though we expected the *PIK3CA* wild-type cell line SW480 to demonstrate less growth suppression, inhibition of CRC cell growth and survival was observed across all cell lines, regardless of mutation status. The cell line DLD1, however, which notably harbors two *PIK3CA* mutations, demonstrated the highest synergy based on CI scores (Figure 1B). This suggests that PI3Kα does indeed play a significant role in CRC cell growth, making inavolisib an effective therapy in the setting of *PIK3CA*-mutated CRC. Despite DLD1 demonstrating the highest synergy scores, this cell line required higher drug concentrations to reach its IC_50_ and to achieve growth suppression in the CFU assay compared to the other four cell lines. This suggests reduced potency of the celecoxib–inavolisib combination treatment in DLD1. SW480 and DLD1 cell lines were cultured as 3D spheroids to explore whether drug effects would vary across 2D and 3D cell culture. As seen in 2D cell culture, growth suppression was observed in both cell lines, regardless of *PIK3CA* mutation status (Figure 2A). While DLD1 and SW480 had similar IC_50_ values for combination treatment in 2D cell culture, the IC_50_ in spheroids was lower for DLD1 than for SW480. Also deviating from the results of 2D cell culture, DLD1 showed synergy at two out of nine drug concentrations as compared to six out of nine for SW480 (Figure 2B).

In our study, when examining CRC cell lines SW480 (*PIK3CA* wild-type) and DLD 1 (*PIK3CA* mutant), we found differing results upon treatment with celecoxib–inavolisib combination therapy. In 2D cell culture, there was similar drug sensitivity to combination treatment, but greater synergy in the *PIK3CA* mutant. In 3D cell culture, combination treatment showed greater synergy in the *PIK3CA* wild-type, but greater sensitivity in the *PIK3CA* mutant. The preclinical assessment of combination cancer therapies must consider both synergy and potency, keeping in mind the implication of dosing and toxicity for future clinical exploration. It is also important to consider the three different cell culture models used in our study: 2D cell culture, 3D cell culture (spheroids), and PDOs. The latter two are 3D but also differ in their composition and structure. 2D cell culture was developed in the early 1900s and has since been the most commonly used culture method due to its time efficiency, simplicity of culture, reproducibility, and low cost. Given the nature of the monolayer, however, this model lacks the same structure, heterogeneity, and variable exposure to both drugs and nutrients seen in tumor tissues [16]. Multicellular tumor spheroids (MCTS) can be cultured from cancer cell lines, as seen in our study. Though they do not histologically resemble original tumor tissue, their 3D structure allows for cell–cell interactions and can mimic metabolic the gradient of a poorly vascularized tumor [17]. As such, they are superior to 2D cell culture in modeling drug efficacy and resistance. Meanwhile, PDOs have become more commonly used in recent years due to advances in culturing techniques and the recognition that they are more faithful representations of tumor heterogeneity and 3D structure than cell lines. Overall, our study reveals that all three types of CRC models were able to demonstrate drug synergy between celecoxib and inavolisib, although there was variation in the potency and depth of synergy that was demonstrated in different models.

To understand the molecular basis of the observed synergistic effects, we assessed the expression of PI3K and MAPK pathway components with Western blot analysis (Figure 3A). The effects of celecoxib–inavolisib combination therapy were examined in all five CRC cell lines grown in 2D culture (Figure 3B) while the effects of celecoxib and inavolisib monotherapy were examined in SW480 and DLD1 (Figure 3C). Though total AKT protein expression did not demonstrate any time-dependent decrease after combination treatment, AKT phosphorylation progressively decreased in all five cell lines (Figure 3B). The lack of decrease in AKT expression may be explained by the mechanism of action of inavolisib, which directly blocks PIP3 activity, subsequently reducing AKT phosphorylation. While inavolisib works to degrade mutated PI3Kα protein, its downstream effects on AKT phosphorylation do not affect levels of AKT itself [8]. As seen with AKT expression, ERK expression was not decreased after combination treatment. ERK phosphorylation responded differently across cell lines following combination treatment: HT29 and DLD1 showed a progressive decrease in p-ERK levels, whereas HCT116 and RKO exhibited a transient reduction followed by rebound elevation. Monotherapy treatment similarly yielded varying results in ERK phosphorylation with SW480 demonstrating an initial decrease followed by rebound elevation in both monotherapy conditions and DLD1 demonstrating a progressive decrease in both monotherapy conditions. Despite these differences, all five cell lines displayed similar synergistic decreases in cell viability, suggesting that suppression of ERK phosphorylation is not essential for the anti-proliferative effects observed.

Additional Western blots were performed on lysate from 3D spheroids grown from SW480 and DLD1 after treatment with celecoxib and inavolisib monotherapy as well as celecoxib–inavolisib combination therapy (Figure 4A). The most notable, consistent pattern seen in the results was the decrease in AKT phosphorylation with inavolisib monotherapy and combination therapy in both cell lines (Figure 4B), also reflected in the p-AKT/AKT ratio (Figure 4C). This effect was present in both 2D and 3D cell culture, strengthening its potential role in the synergistic mechanism of celecoxib–inavolisib combination therapy. While inavolisib monotherapy also demonstrated suppression of AKT phosphorylation in the Western blots, in both the 2D and 3D cell drugging experiments, inavolisib had a noticeably higher IC_50_ than combination therapy across all cell lines. This possibly suggests increased effectiveness and synergy of drug treatment with the addition of celecoxib (Figure 1A and Figure 2A).

The decreased levels of mitogenic signaling marker p-AKT seen in all 2D and 3D cell line Western blots suggests a growth suppression effect in CRC cells treated with celecoxib–inavolisib combination therapy. These results, however, were not reflected in PDO IF staining. Values of Ki-67 activity were analyzed by one-way ANOVA assuming Gaussian distribution and, where appropriate, Šídák’s test and Dunnett’s test were applied for multiple comparisons (Supplemental Figure S2A). For both *PIK3CA* wild-type CK 10278 and *PIK3CA* mutant CRC 23-006 Dunnett’s test showed no significant difference in percentage of cells with positive Ki-67 staining in any drug condition as compared to control. Šídák’s test for both PDO models also showed no significant difference in between drug treatment groups. This may be explained when considering the levels of p-ERK, another marker of mitogenic signaling, after combination treatment. While there was no clear trend across the cell line Western blots, p-ERK levels showed a rebound increase in HCT116 and RKO. In the other three cell lines, p-ERK levels, while decreased, were still present at higher levels as compared to corresponding p-AKT levels at nearly all time points (Supplemental Figure S1A). The same held true for the 3D spheroids Western blots, with no distinct trend in p-ERK levels, but higher levels of p-ERK relative to p-AKT (Supplemental Figure S1B). Both the PI3K/AKT and MAPK/ERK pathways play roles in cell growth, proliferation, and survival and share a significant amount of functional overlap. Moreover, these pathways share signaling nodes such as Ras and PIP_2_, allowing for crosstalk and compensatory activation in the setting of inhibition of one of the pathways, as in the case of treatment with inavolisib [18]. As such, compensation from MAPK/ERK pathway activation is a plausible explanation for the discrepancies between p-AKT levels in cell line Western blots and Ki-67 expression in IF staining of PDOs. In the future, dual inhibition of PI3K and MAPK pathways could potentially be tested as a therapeutic approach.

Values of Cas-3 activity were analyzed using the same statistical methods (Supplemental Figure S2B). For CK 10128 Dunnett’s test showed a significantly higher percentage of cells with positive Cas-3 staining in the inavolisib monotherapy and combination therapy groups as compared to control, respectively (*p* = 0.045, *p* = 0.0306). Šídák’s test, however, showed no significant difference between drug treatment groups. For CRC 23-006 multiple comparisons with Dunnett’s and Šídák’s tests showed no significant differences between control and drug conditions, nor between various drug treatment groups. The lack of statistically significant data from our IF experiments as a whole likely stem from the variability of the assay used—in addition to size and growth discrepancies inherent to the two different PDO models, inconsistencies in cell staining required manual counting and analysis, introducing another degree of variability.

Apoptotic pathways were also examined in CRC cell lines by assessing Annexin V and PS staining in *PIK3CA* wild-type SW480 and *PIK3CA* mutant DLD1, with results quantified as percentage of apoptotic cells (Figure 7B). These values were analyzed by one-way ANOVA assuming Gaussian distribution and, where appropriate, Šídák’s test and Dunnett’s test were applied for multiple comparisons. For SW480 multiple comparisons with Dunnett’s test showed a significant difference in percentage of apoptotic cells for all drug conditions when compared to control. However, multiple comparisons with Šídák’s test showed no significant difference in between various drug conditions. For DLD1 multiple comparisons with Dunnett’s test showed that combination therapy was the only drug condition with a significantly higher percentage of apoptotic cells as compared to control (*p* = 0.0021). Multiple comparisons with Šídák’s test also showed that combination therapy had a significantly higher percentage of apoptotic cells when compared to celecoxib and inavolisib monotherapy, respectively (*p* = 0.0218, *p* = 0.0039). This suggests a higher degree of apoptosis induced by combination therapy which was only seen in the *PIK3CA* mutant cell line DLD1. These results echo those of Western blots performed on 3D spheroids, in which PARP cleavage for DLD1 showed a time-dependent increase after combination treatment, notably greater than the modest increase seen for SW480. Though the results of the cell line apoptosis assay were not consistent with those of Cas-3 staining in PDOs, they may be considered more reliable based on the robustness of the apoptosis assay in comparison to the aforementioned issues with the IF experiments.

While *PIK3CA* mutational status did not play a significant role in CRC cell line response to the celecoxib–inavolisib combination therapy, this was not reflected in PDOs. *PIK3CA* mutant CRC 23-006 showed markedly lower IC_50_ values for inavolisib monotherapy and combination therapy in comparison to *PIK3CA* wild-type CK 10278 (Figure 5A). It also showed greater synergy, as seen in the CI scores (Figure 5B). The stark difference in efficacy of both inavolisib monotherapy and combination therapy against CRC 23-006 as compared to celecoxib monotherapy further supports the role of *PIK3CA* mutation in the tumorigenesis of CRC. The discrepancy between cell line and PDO results could potentially be explained by mutations outside of *PIK3CA*. Cell lines HCT116 and DLD1 both harbor *KRAS* G13D mutations, while RKO harbors a *BRAF* V600E mutation [19,20,21,22,23]. In the setting of PI3Kα inhibition and a resulting decrease in AKT activation, the *KRAS* G13D mutation can act as a resistance mechanism by maintaining downstream AKT activation in addition to activating ERK [24]. The *BRAF* V600E mutation can similarly serve as a resistance mechanism by driving constitutive MAPK activation, independent of upstream PI3K or Ras signaling [25]. Although whole exome sequencing of CRC 23-006 indicated a *KRAS* A146T mutation, it is associated with a less active phenotype than more common *KRAS* mutations such as G12D or G13D, potentially making it a less effective mechanism of resistance against PI3Kα inhibition [26]. Notably, next-generation sequencing of CK 10278 indicated a *KRAS* G12D mutation which could further explain the discrepancies in the results of the PDO drugging and IF staining experiments when compared to CRC 23-006 [27]. In addition to these mutational differences, we should again consider the different cell culture models used in this study. In comparison to 2D and 3D cell culture, PDOs are established from actual tumor tissues, allowing for preserved heterogeneity and more complex structure reminiscent of the organ of origin [28]. After treatment with combination therapy the *PIK3CA* mutant cell line in 2D cell culture showed greater synergy but not sensitivity, while in 3D culture it showed greater sensitivity but not synergy. When examining PDOs, however, the *PIK3CA* mutant model demonstrated considerably greater drug sensitivity and synergy as compared to *PIK3CA* wild-type model. Given the structural and compositional differences between these various models, the drugging results from our PDO experiment likely hold the most clinical relevance.

Several limitations of our study are worth noting. First, our mechanistic findings warrant further investigation through the study of additional CRC models and functional experiments such as genetic knockdown. The IF staining experiments were intended to evaluate the mechanism behind the synergistic effects of celecoxib–inavolisib combination treatment; however, we were unable to collect statistically significant data, largely due to the degree of variability in our assay. Second, our combination results should be confirmed using in vivo models prior to advancing to clinical testing. Finally, a more thorough analysis of markers of resistance and sensitivity as well as potential compensatory or resistance mechanisms would help accelerate the translation of this combination concept.

## 5. Conclusions

In summary, our study demonstrates that combination therapy with celecoxib, a selective COX-2 inhibitor, and inavolisib, a selective PI3Kα inhibitor, exerts synergistic growth inhibition across multiple CRC cell lines and PDOs with varying *PIK3CA* mutation statuses, potentially through suppression of AKT phosphorylation. A significant increase in cellular apoptosis was also seen with combination therapy as compared to monotherapy, present only in the setting of *PIK3CA* mutation. A key strength of our study is the inclusion of a diverse range of CRC cell lines grown in both 2D and 3D cell culture, as well as PDOs, enhancing the physiological relevance of our findings. Additionally, inclusion of PDO CRC 23-006 added to the breadth of our results, as it represents a genomic background not readily available among established CRC cell lines, with a *PIK3CA* mutation and a weakly activating *KRAS* mutation, comparable to a *KRAS* wild-type context. This study, however, is limited since only two PDO models were used. Future studies could include a wider range of models to assess different *PIK3CA* mutations or dual *PIK3CA* mutations, as seen in DLD1, as well as in vivo models. In our study, we were not yet able to delineate a simple monogenetic biomarker, such as *PIK3CA* mutation, that could reliably predict response to treatment. This is likely due to the overall genomic and biological complexity of CRC, as well as the presence of additional well-characterized co-occurring driver mutations, such as *KRAS* and *BRAF*. Thus, a more exploratory approach for biomarkers during early phase trials may be the most prudent, so as not to eliminate entire categories of patients during the early phases of clinical investigation. Ultimately, our findings provide preliminary support for celecoxib–inavolisib combination treatment as a rational therapeutic avenue warranting further preclinical investigation.

## Data Availability

All data in this manuscript are available upon reasonable request. Genomic and transcriptomic data will be submitted to appropriate databases once the manuscript is in press. Patient-derived organoids will be submitted to appropriate biobanks once the manuscript is in press.

## Supplementary Materials

The following supporting information can be downloaded at: https://www.mdpi.com/article/10.3390/pharmaceutics18060683/s1, Figure S1: (A) Quantification of results of combination therapy cell line Western blots expressed as optical density (OD) normalized to ß-actin. (B) Quantification of results of spheroid Western blots expressed as optical density (OD) normalized to ß-actin; Figure S2: (A) Quantification of Ki-67 activity in patient-derived organoids 48 h after drugging with single-agent celecoxib, inavolisib, and celecoxib/inavolisib combination treatment expressed as percentage of positively stained cells. All *p*-values from multiple comparisons using Dunnett’s and Šídák’s tests are listed at right. (B) Quantification of Cas-3 activity in patient-derived organoids 48 h after drugging with single-agent celecoxib, inavolisib, and celecoxib/inavolisib combination treatment expressed as percentage of positively stained cells. All *p*-values from multiple comparisons using Dunnett’s and Šídák’s tests are listed at right (significant *p*-values are indicated in bold); Table S1: Table of ATCC numbers for cell lines used; Table S2: Table of antibodies cited in Materials and Methods.
