# Supplementary material for: Synergistic Inhibition of Colorectal Cancer Growth by Combined PI3K and COX-2 Blockade in Cell Lines and Patient-Derived Organoids
Source: Pharmaceutics. 2026 May 30;18(6):683. doi: 10.3390/pharmaceutics18060683 (PMC13305696; doi:10.3390/pharmaceutics18060683)
Supplement: Supplementary file 1 [file pharmaceutics-18-00683-s001.zip › pharmaceutics-3979578-supplementary.pdf]

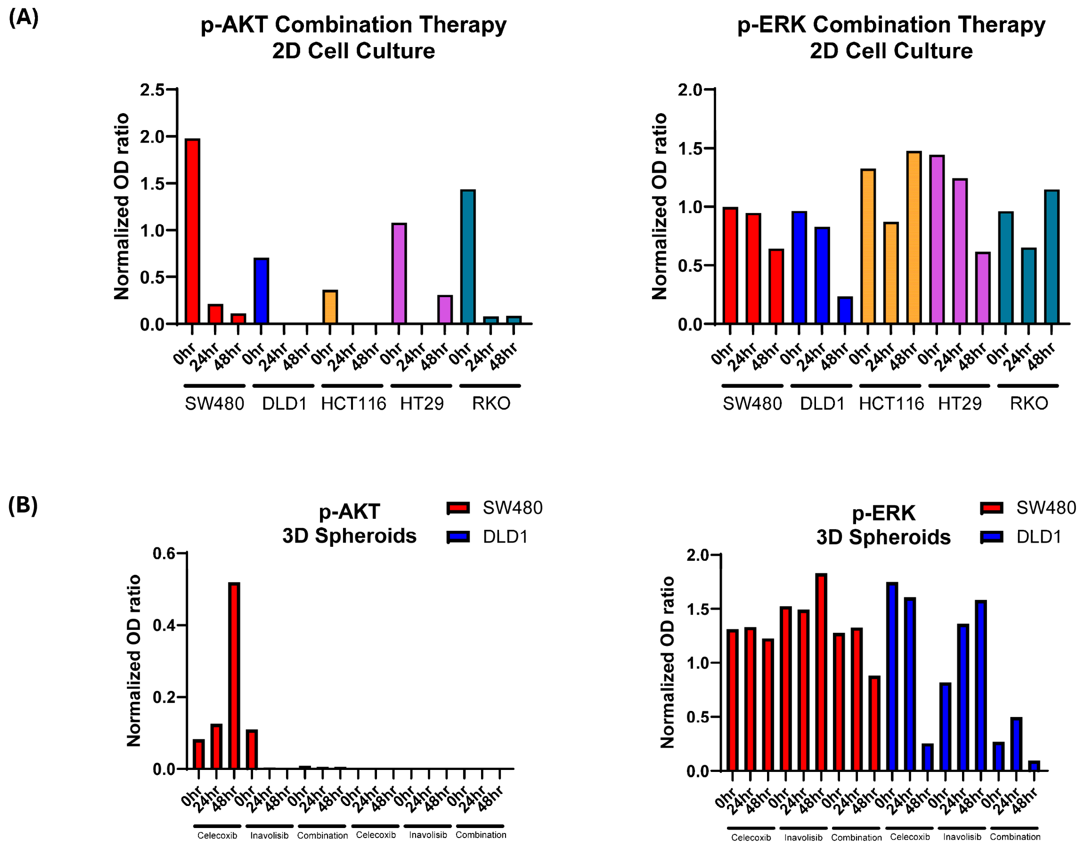

Figure S1: (A) Quantification of results of combination therapy cell line Western blots expressed as optical density (OD) normalized to ß-actin. (B) Quantification of results of spheroid Western blots expressed as optical density (OD) normalized to ß-actin

(A)

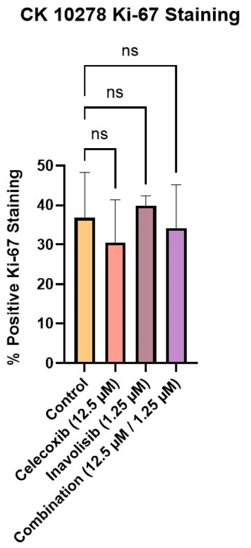

Dunnett’s Test
Control vs. Celecoxib
p = 0.7645

Control vs. Inavolisib
p = 0.9614

Control vs. Combination
p = 0.9718

Šídák’s Test
Combination vs. Celecoxib
p = 0.9983

Combination vs. Inavolisib
p = 0.9818

Celecoxib vs. Inavolisib
p = 0.846

* p < 0.05
** p < 0.01
*** p < 0.001
ns – not significant

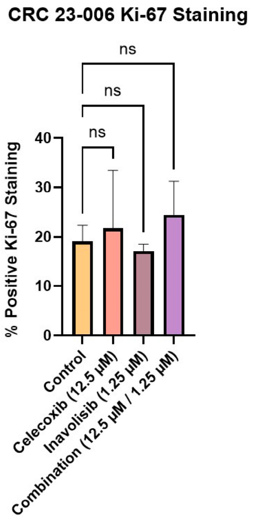

Dunnett’s Test
Control vs. Celecoxib
p = 0.9363

Control vs. Inavolisib
p = 0.9673

Control vs. Combination
p = 0.6796

Šídák’s Test
Combination vs. Celecoxib
p = 0.998

Combination vs. Inavolisib
p = 0.7887

Celecoxib vs. Inavolisib
p = 0.9666

* p < 0.05
** p < 0.01
*** p < 0.001
ns – not significant

(B)

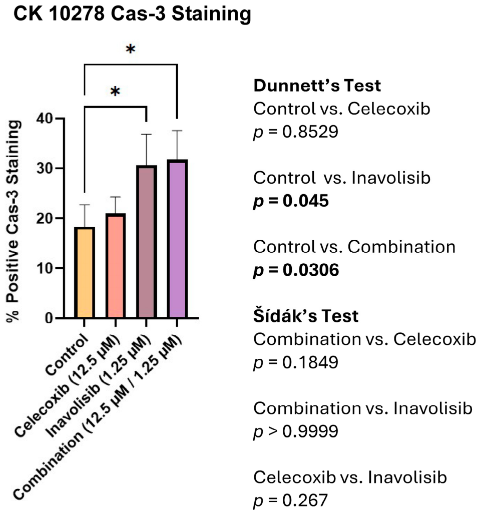

Dunnett’s Test
Control vs. Celecoxib
p = 0.8529

Control vs. Inavolisib
***p* = 0.045**

Control vs. Combination
***p* = 0.0306**

Šídák’s Test
Combination vs. Celecoxib
p = 0.1849

Combination vs. Inavolisib
p > 0.9999

Celecoxib vs. Inavolisib
p = 0.267

* p < 0.05
** p < 0.01
*** p < 0.001
ns – not significant

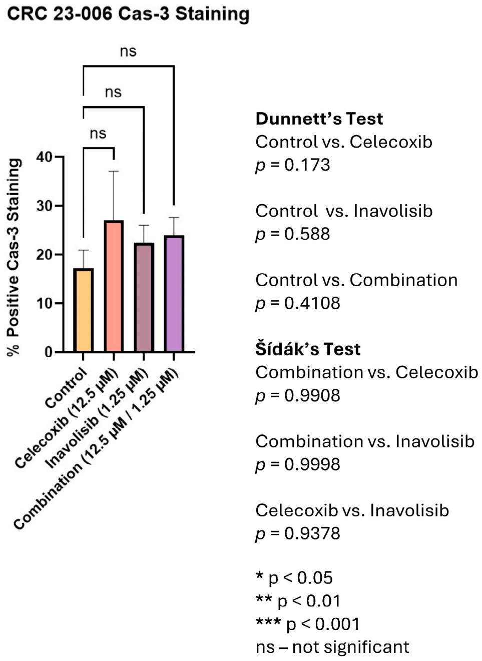

Dunnett’s Test
Control vs. Celecoxib
p = 0.173

Control vs. Inavolisib
p = 0.588

Control vs. Combination
p = 0.4108

Šídák’s Test
Combination vs. Celecoxib
p = 0.9908

Combination vs. Inavolisib
p = 0.9998

Celecoxib vs. Inavolisib
p = 0.9378

* p < 0.05
** p < 0.01
*** p < 0.001
ns – not significant

Figure S2: (A) Quantification of Ki-67 activity in patient-derived organoids 48 h after drugging with single-agent celecoxib, inavolisib, and celecoxib/inavolisib combination treatment expressed as percentage of positively stained cells. All p-values from multiple comparisons using DunneĴ’s and Šídák’s tests are listed at right. (B) Quantification of Cas-3 activity in patient-derived organoids 48 h after drugging with single-agent celecoxib, inavolisib, and celecoxib/inavolisib combination treatment expressed as percentage of positively stained cells. All p-values from multiple comparisons using DunneĴ’s and Šídák’s tests are listed at right (significant p-values are indicated in bold)

Table S1: Table of ATCC numbers for cell lines used

Cell Line	ATCC number
SW480	CCL-228
DLD1	CCL-221
HCT116	CCL-247EMT
HT29	HTB-38
RKO	CRL-2577
L-WRN	CRL-3276

Table S2: Table of antibodies cited in Materials and Methods.

Source	Item Name	Catalogue Number	Dilution	Incubation Temperature/Time
Abcam	Anti-Ki67 antibody	AB15580	1:200	4°C Overnight
Biotium	Norview 488 Caspase-3 Substrate	10402	1:200	4°C Overnight
Cell Signaling	Akt (pan) (C67E7)	4691	1:1000	4°C Overnight
Cell Signaling	Phospho-Akt (Ser473) (D9E) XP®	4060	1:1000	4°C Overnight
Cell Signaling	PI3 Kinase p110α (C73F8)	4249	1:1000	4°C Overnight
Cell Signaling	PARP	9542	1:1000	4°C Overnight
Cell Signaling	Cox2 (D5H5) XP®	12282	1:1000	4°C Overnight
Cell Signaling	p44/42 MAPK (Erk1/2)	9102	1:1000	4°C Overnight
Cell Signaling	Phospho-p44/42 MAPK (Erk1/2) (Thr202/Tyr204)	9101	1:1000	4°C Overnight
Cell Signaling	β-Actin (8H10D10)	3700	1:1000	4°C Overnight
Invitrogen	Stabilized peroxidase conjugated goat Anti-mouse (H+L)	324030	1:5000	RT 1 hour
Biolegend	HRP Donkey Anti-Rabbit IgG	406401	1:5000	RT 1 hour
Invitrogen	Alexa 647 Anti-Rabbit	A32733	1:200	RT 2 hour
